# Teaching Medical Microbiology With a Web-Based Course During the COVID-19 Pandemic: Retrospective Before-and-After Study

**DOI:** 10.2196/39680

**Published:** 2023-02-27

**Authors:** Cihan Papan, Monika Schmitt, Sören L Becker

**Affiliations:** 1 Center for Infectious Diseases Institute of Medical Microbiology and Hygiene Saarland University Homburg Germany; 2 Institute for Hygiene and Public Health University Hospital Bonn Bonn Germany

**Keywords:** SARS-CoV-2, COVID-19, online learning, web-based learning, web-based course, medical students, medical microbiology, microbiology, medical education, medical school, online teaching, online course, online class, online instruction, distance learning, distant learning, performance, student, learning outcome, perception, opinion, attitude, examination, practical course

## Abstract

**Background:**

The COVID-19 pandemic has imposed unprecedented hurdles on health care systems and medical faculties alike. Lecturers of practical courses at medical schools have been confronted with the challenge of transferring knowledge remotely.

**Objective:**

We sought to evaluate the effects of a web-based medical microbiology course on learning outcomes and student perceptions.

**Methods:**

During the summer term of 2020, medical students at Saarland University, Germany, participated in a web-based medical microbiology course. Teaching content comprised clinical scenarios, theoretical knowledge, and instructive videos on microbiological techniques. Test performance, failure rate, and student evaluations, which included open-response items, for the web-based course were compared to those of the on-site course from the summer term of 2019.

**Results:**

Student performance was comparable between both the online-only group and the on-site comparator for both the written exam (n=100 and n=131, respectively; average grade: mean 7.6, SD 1.7 vs mean 7.3, SD 1.8; *P*=.20) and the oral exam (n=86 and n=139, respectively; average grade: mean 33.6, SD 4.9 vs mean 33.4, SD 4.8; *P*=.78). Failure rate did not significantly differ between the online-only group and the comparator group (2/84, 2.4% vs 4/120, 3.3%). While lecturer expertise was rated similarly as high by students in both groups (mean 1.47, SD 0.62 vs mean 1.27, SD 0.55; *P*=.08), students who took the web-based course provided lower scores for interdisciplinarity (mean 1.7, SD 0.73 vs mean 2.53, SD 1.19; *P*<.001), opportunities for interaction (mean 1.46, SD 0.67 vs mean 2.91, SD 1.03; *P*<.001), and the extent to which the educational objectives were defined (mean 1.61, SD 0.76 vs mean 3.41, SD 0.95; *P*<.001). Main critiques formulated within the open-response items concerned organizational deficits.

**Conclusions:**

Web-based courses in medical microbiology are a feasible teaching option, especially in the setting of a pandemic, leading to similar test performances in comparison to on-site courses. The lack of interaction and the sustainability of acquired manual skills warrant further research.

## Introduction

The COVID-19 pandemic, caused by the SARS-CoV-2 virus, is arguably one of the biggest crises of modern times, with a multitude of repercussions on societal, economic, and medical systems [1-4]. A considerable fallout has affected school and university education alike [5]. In many countries, primary and secondary schools were closed during the first pandemic wave in spring 2020 [6-8], and the majority of universities were equally overwhelmed by this inciting incident [9]. Without a ready-made alternative plan, medical faculties suspended on-site education and were forced to hastily provide provisional materials via web-based platforms [10]. While the theoretical content of preclinical courses can be regarded as more easily adaptable to an online format, lecturers of practical courses, such as dissection or microscopy courses, struggle substantially to remotely present knowledge and manual skills [11-13]. As such, medical (or clinical) microbiology is a subject containing both theoretical knowledge and practical skills. Moreover, it is a subject that is not only critical for diagnostic purposes but is also important for understanding diseases caused by emerging pathogens such as bacteria, fungi, or viruses. Thus, it carries an inherent importance for medical students and, hence, future physicians, especially in the face of future potential pandemics and the already prevalent shortage in microbiologists and infectious disease specialists [14,15].

Although some literature on adapted medical education has cumulated since the beginning of the pandemic [16-20], data on the specific hurdles to implementing online or distant learning in medical microbiology during the COVID-19 pandemic are scarce. Particular challenges that could threaten the quality of online learning include technical difficulties, reduced social interactions, “video-conferencing fatigue,” and lack of focus among learners [21]. Some of these challenges touch upon “transactional distance,” which occurs between a student and a faculty member when interacting through a technological platform [22]. According to the Theory of Transactional Distance by Moore [23], learners in an online format experience particular interactions that not only include the faculty, other learners, and the subject matter, but also the delivery platform itself and external resources. However, with an adequate design and delivery strategy, online learning tools can overcome these hurdles [24]. Previously, additional online learning for medical microbiology had been shown to be beneficial for student performance in a before-and-after study from Dublin in the prepandemic era [25]. Here, we sought to evaluate the effectiveness of a web-based microbiology course compared with an on-site course format by measuring exam results and student perceptions at a single center in Germany during the first wave of the COVID-19 pandemic in 2020. We hypothesized that student performance and satisfaction would be comparable between the web-based and on-site course formats.

## Methods

### Study Design

During the summer term of 2020 (April to July 2020), medical students at Saarland University, Germany, participated in a novel, web-based course in medical microbiology, delivered via a modular object–oriented dynamic learning environment (Moodle). Teaching content comprised lectures with audio recordings; clinical scenarios, including high-resolution imaging of agar plates and Gram stains; and instructive videos on microbiological techniques (see Figures S1-S4 in Multimedia Appendix 1). Techniques that were video-captured included a Gram staining; catalase, coagulase, and oxidase tests; and streak and spread plating. Photographs and videos were captured with a Panasonic Lumix DMC GH4 (Panasonic Corporation) and a Sigma 18-35 mm f/1.8 lens (Sigma Corporation), adapted with an MFT T Speed Booster XL (Metabones). Videos were edited with iMovie (Apple Inc).

Students’ test performance, failure rate, and perceptions and satisfaction pertaining to the web-based course were compared to those of the students who took the on-site course in the 2019 summer term. Both cohorts were at the same time point in terms of the progression of their studies when starting their respective course.

### Examinations

The written exam was performed on paper and in person. It consisted of 10 multiple-choice or open-item questions, covering the topics of medical microbiology, infectious diseases, infection prevention and control, and vaccinations (maximum of 10 points). The in-person oral exam included questions on 5 thematic complexes from the domains described above (maximum of 40 points). In addition, a written exam on virology had to be taken as well (maximum of 10 points). In total, the pass/fail score was ≥60% (36 out of 60 points). Students can choose to postpone either the written or oral exam to a later time point or term. To assess the failure rate, only students who took both the written and oral exams were taken into account.

### Student Evaluation

Course evaluation by the students was assessed using a 5-point Likert scale and open-text questions via a web-based platform. Invitations were distributed via email. The open-text answers from the students of the web-based course were analyzed in terms of their predominant value, either positive or negative, and simultaneously grouped into the following domains: interaction between students and faculty, practical content of the course, organizational aspects, and quality of content.

### Statistics

Statistical analyses were performed with GraphPad Prism (version 8.0; GraphPad Software Inc), using a 2-sided *t* test for continuous variables and the Fisher exact test for categorical data. Using the Bonferroni correction in light of multiple testing needed for the 9 items obtained in the course evaluation, we calculated and set the statistical significance level at .0056 (.05/9).

### Ethical Considerations

All data were obtained during the provision of student education. All data analyses were carried out in accordance with relevant regulations. No administrative permissions were required to access the raw data used in this study. Course evaluation by students was conducted anonymously and voluntarily. All data used in this study were completely anonymized. In addition, quantitative data were obtained as an aggregated data set. Since no individual, identifiable student data, including biomedical, clinical, and biometric data, were used, neither ethical committee approval nor informed consent was necessary.

## Results

### Exam Results

**Figure 1:**
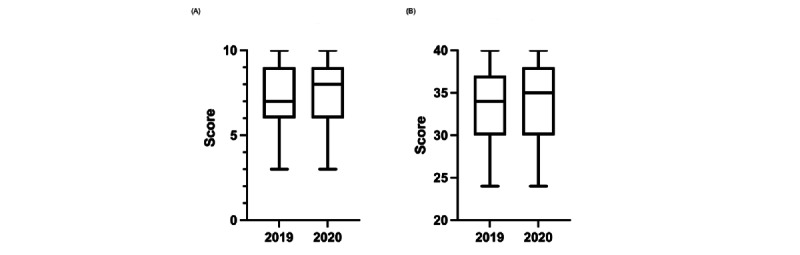
Whisker plots of the (A) written and (B) oral exam results for students who took the on-site course (2019) and the web-based course (2020).

In the web-based course, 100 students took the written exam, 86 took the oral exam, and 84 took both exams. Of the students in the on-site course, 139 took the oral exam, 131 took the written exam, and 120 took both exams. The mean score for the written exam was 7.6 (SD 1.7; median 8, 95% CI 6-9) for the web-based course and 7.3 (SD 1.8; median 7, 95% CI 6-9) for the on-site course (*P*=.20) (Figure 1). The mean score of the oral exam was 33.6 (SD 4.9; median 35, 95% CI 30-38) for the web-based course and 33.3 (SD 4.8; median 34, 95% CI 30-37) for the on-site course (*P*=.73) (Figure 1).

There was no significant difference in the failure rate between students in both years. In the online-only group, 2 out of 84 students failed the exam (failure rate of 2.4%), compared to 4 out of 120 students in the on-site course (failure rate of 3.3%) (*P*≥.99).

### Evaluation Results

**Figure 2:**
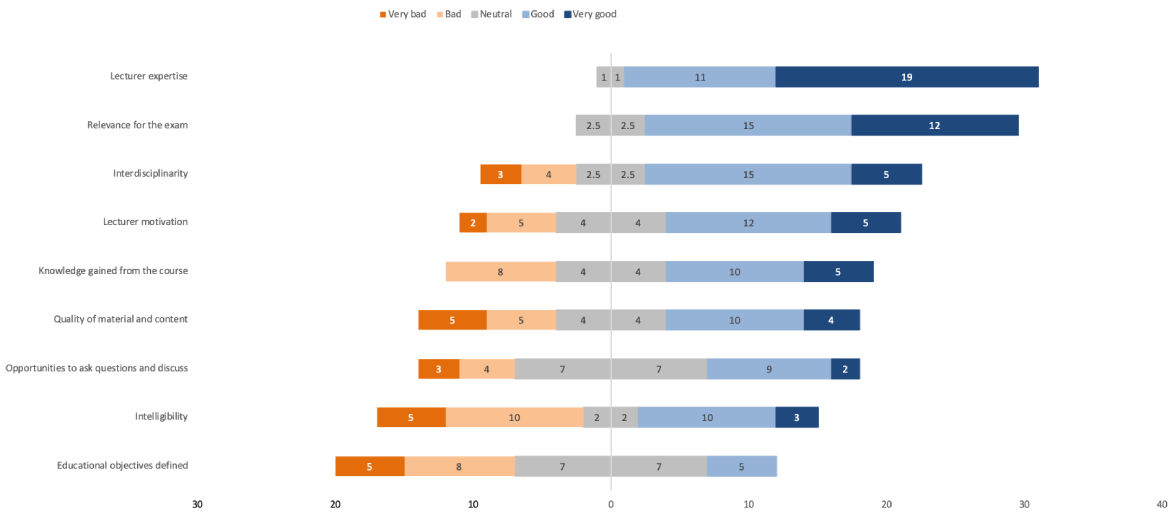
Distribution of the online-only students’ rating of the different aspects of the course on a 5-point Likert scale, from “very bad” (dark orange) to “very good” (dark blue).

**Figure 3:**
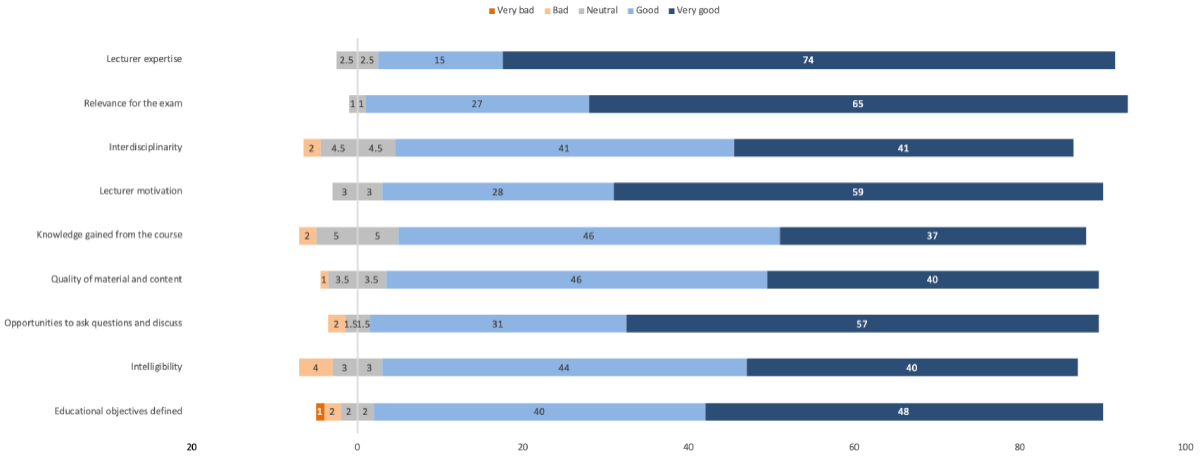
Distribution of the on-site course students’ rating of the different aspects of the course on a 5-point Likert scale, from “very bad” (dark orange) to “very good” (dark blue).

The evaluation was completed by 96 and 32 students for the on-site and web-based courses, respectively. While lecturer expertise was rated similarly as high by students in both groups, students from the online-only group provided lower scores for the course’s relevance for the exam, its level of interdisciplinarity, the motivation of the lecturer, and the knowledge they gained from the course (Table 1; Figures 2 and 3).

Differences were more distinct for the following aspects: quality of the course material and content, opportunities to ask questions and for discussion, intelligibility and clarity, and the extent to which the educational objectives were defined (Table 1; Figures 2 and 3). We asked for the level of challenge posed by the course as perceived by the students; while a similar proportion of students in both the web-based course and the on-site course regarded the educational challenge of their respective course as adequate (18/31, 58% vs 56/94, 60%; *P*≥.99), 19 out of 31 (61%) students from the online-only group stated that they would recommend the course compared to 73 out of 90 (81%) students in the on-site course (*P*=.049).

The main critique concerned organizational aspects (32 negative mentions vs 1 positive mention), including the overlap of exam dates with other subjects, delivery of information and content on short notice, and time constraints with regard to the exam preparation period. Furthermore, the lack of practice was criticized (2 negative mentions), although it was acknowledged that this was due to the special circumstances. Of note, the opportunities for interaction were rated predominantly positively (2 negative vs 5 positive mentions) in the open-text answers. Similarly, the quality of the content received 10 negative and 22 positive remarks. We specifically analyzed mentions of the unique multimedia content, identifying 16 additional positive mentions.

**Table 1:** Mean (SD) scores (1=very good, 2=good, 3=moderate, 4=weak, 5=very weak) for different items of the evaluation completed by students in the on-site course (2019) and the web-based course (2020).

Item	On-site course (n=96), mean (SD)	Web-based course (n=32), mean (SD)	*P* value
Grade the expertise of the lecturer.	1.27 (0.55)	1.47 (0.62)	.08
To what extent do you regard the course as relevant to the exam?	1.33 (0.52)	1.78 (0.70)	<.001
Grade the level of interdisciplinarity.	1.7 (0.73)	2.53 (1.19)	<.001
Grade the motivation of the lecturer.	1.43 (0.61)	2.59 (1.13)	<.001
Grade the knowledge gained from the course.	1.66 (0.77)	2.61 (1.05)	<.001
Grade the quality of the course material and content.	1.71 (0.8)	2.91 (1.28)	<.001
Grade the opportunities provided to ask questions and for discussion.	1.46 (0.67)	2.91 (1.03)	<.001
To what extent was the course intelligible and clear?	1.7 (0.77)	3.13 (1.29)	<.001
How well were the educational objectives defined?	1.61 (0.76)	3.41 (0.95)	<.001

## Discussion

### Principal Findings

The undisputed challenges posed by the COVID-19 pandemic demanded quick and feasible solutions for students of all levels and subjects on a global scale. In this before-and-after study performed in real-life pandemic circumstances, we showed that a web-based medical microbiology course for undergraduates led to similar learning outcomes, as measured by exam results, to a conventional, on-site course, even though several aspects of the web-based course were evaluated with significantly lower scores by the students. In addition, the web-based course was met with discontent owing to mainly organizational drawbacks.

Similarly, in a survey study from California by Shahrvini and colleagues [26], medical undergraduates, despite appreciating this more flexible way of learning, still perceived preclinical remote learning as disadvantageous due to the lack of opportunities for participation. Of note, this study revealed that the quality of instruction is a recurrent issue, as observed in our study, that merits further attention in order to improve distant learning experiences.

Depending on the geographical background of students, other challenges may also be prevalent, such as technical, infrastructural, or financial issues [27]. As shown previously [25], online elements can be beneficial for student performance in fields outside of medical microbiology; however, students have reported being in favor of a blended approach that combines the advantages of both self-paced online learning and in-person instruction in a lab environment [28].

### Strengths and Limitations

Our study has several strengths. To the best of our knowledge, this is the first study to assess the hurdles faced by a medical microbiology faculty during the COVID-19 pandemic and the feasibility of a web-based teaching alternative while simultaneously monitoring the transition from in-person to online teaching formats. Furthermore, our approach contained an in-depth qualitative analysis of students’ perceptions, which may help to deliver improved undergraduate education in the terms to come. This is especially true since further restrictions on on-site teaching are to be expected due to the presence and increasing predominance of SARS-CoV-2 variants of concern with increased transmissibility [29] and the somewhat slow rollout of mass vaccinations [30].

Our study also has limitations. First, this is a single-center experience from one country, which may limit its generalizability. Second, the noninferior exam results during the pandemic term may have been influenced by a more generous approach taken by the examiners than in the previous year, owing to an inherent understanding of the difficult situation. Third, we analyzed the summer term of 2020, which already dates back several terms, while modes and methods of online learning have rapidly evolved since the beginning of the pandemic. Hence, even more modern technologies are available and used in both undergraduate and postgraduate teaching [31-35]. Furthermore, course evaluation by the students was voluntary, leading to a smaller number of respondents than students taking the respective exams. Another limitation is the fact that students could postpone the exam, which may have biased the results of the online-only cohort as some students may have been struggling with the new format. Last, but not least, it has to be acknowledged that the course duration and hence the content had to be reduced, and although the multimedia content was appreciated, manual skills cannot be completely substituted by web-based learning alone.

The acceptance of or resistance to online learning, in general, may partly be subject to generational influences as well. Students in 2020 and 2021 could presumably be more open, acquainted, and comfortable with (social) media as a platform for knowledge transfer and dissemination than students from previous decades [33,36-38].

The findings of our study are relevant for faculties and decision makers in medical education, primarily in, but not limited to, medical microbiology, as shown previously for other subjects as well (eg, virtual microscopy courses in histology [39]). Despite its largely devastating effects, the pandemic can be seen as a “catalyst of change” that also incited innovation, especially pertaining to (digital) education [40]. Novel technologies will continue to be introduced into medical education and ideally will facilitate the delivery of practical course content in online formats [41-45].

### Conclusions

We showed that web-based undergraduate teaching in medical microbiology is partly feasible with the right tools, but efforts must be made to circumvent subpar organization, lack of face-to-face interaction, and limited opportunities for participation. Additionally, the lack of skills training is an undeniable issue that needs further focus, especially for subjects with practical content. With the unpredictable nature of the pandemic, it is highly conceivable that adaptations to medical curricula will be required both in the short and medium terms. Future studies should therefore focus on identifying the correct balance between online and on-site training, as well as evaluating the utility of novel tools and formats such as mobile phone apps, while also avoiding a lack of constructive alignment that can accrue due to the differences between the mode of teaching and the mode of assessment.

## Appendix

Multimedia Appendix 1 Sample teaching content.

## Data Availability

The data sets used and analyzed in this study are available from the corresponding author upon reasonable request.
