# Supplementary material for: Teaching Medical Microbiology With a Web-Based Course During the COVID-19 Pandemic: Retrospective Before-and-After Study
Source: JMIR Med Educ. 2023 Feb 27;9:e39680. doi: 10.2196/39680 (PMC10012015; doi:10.2196/39680)
Supplement: Multimedia Appendix 1 [file mededu_v9i1e39680_app1.docx]

Supplement to:

Papan et al., Teaching medical microbiology with an online course during the COVID-19 pandemic: a retrospective before and after study


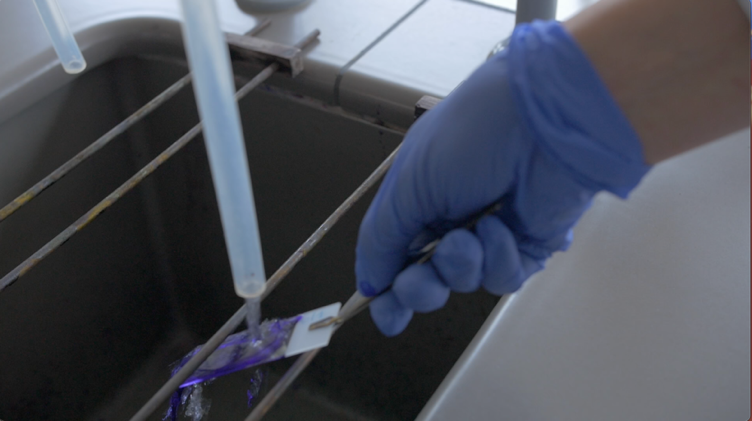


**Figure S1.** Video still of Gram staining.


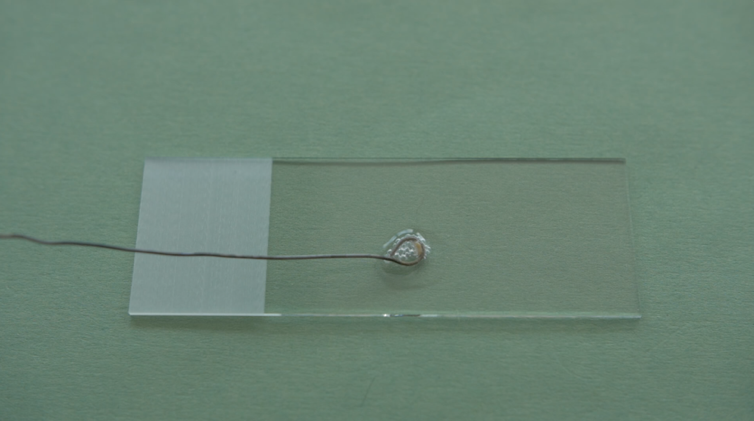


**Figure S2.** Video still of catalase test.


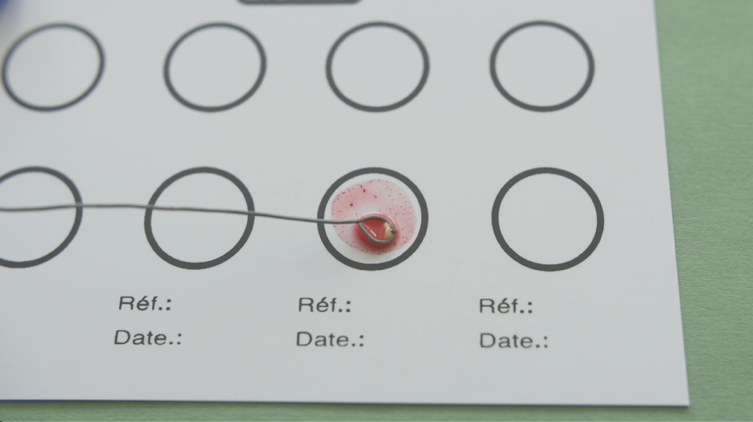


**Figure S3.** Video still of coagulase test.


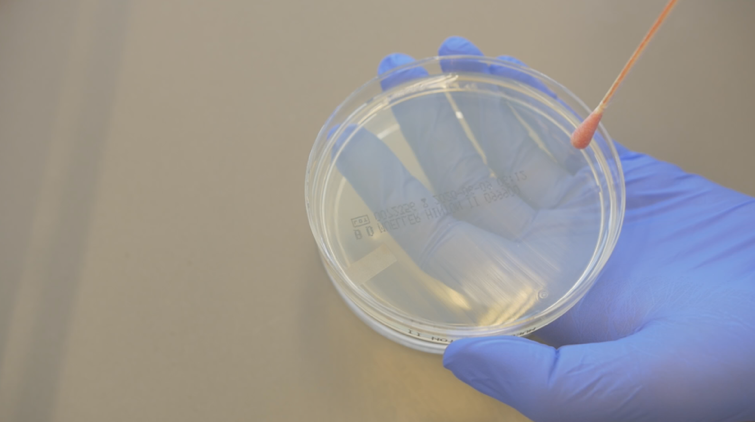


**Figure S4.** Video still of spread plating
